# Evaluation of a Technology-Based Peer-Support Intervention Program for Preventing Postnatal Depression (Part 1): Randomized Controlled Trial

**DOI:** 10.2196/12410

**Published:** 2019-08-29

**Authors:** Shefaly Shorey, Cornelia Yin Ing Chee, Esperanza Debby Ng, Ying Lau, Cindy-Lee Dennis, Yiong Huak Chan

**Affiliations:** 1 Alice Lee Centre for Nursing Studies Yong Loo Lin School Of Medicine National University of Singapore Singapore Singapore; 2 Department of Psychological Medicine University Medicine Cluster National University Hospital Singapore Singapore; 3 Division of Medical Oncology and Hematology Department of Medicine University of Toronto Toronto, ON Canada

**Keywords:** anxiety, loneliness, postpartum depression, social support, technology, digital health, peer support, peer-to-peer support, online support groups, internet

## Abstract

**Background:**

The frenzy of postbirth events often takes a toll on mothers’ mental well-being, leaving them susceptible to postpartum psychological disorders such as postnatal depression (PND). Social support has been found to be effective in restoring the emotional well-being of new mothers. Therefore, mothers need to be supported during the crucial postpartum period to buffer the negative after effects of childbirth and to promote healthier maternal well-being.

**Objective:**

This study aimed to evaluate the effectiveness of a technology-based peer-support intervention program (PIP) on maternal outcomes during the early postpartum period.

**Methods:**

A randomized, parallel-armed controlled trial was conducted. The study recruited 138 mothers (69 in intervention group, 69 in control group) at risk of PND from a tertiary hospital in Singapore. To support these mothers, 20 peer volunteers were recruited by word of mouth and trained by a psychiatrist in social support skills before the intervention commenced. The 4-week–long intervention included a weekly follow-up with a peer volunteer through phone calls or text messages. The intervention group received peer support in addition to the standard care offered by the hospital. The control group only received postnatal standard care. Maternal outcomes (PND, postnatal anxiety [PNA], loneliness, and perceived social support) were measured with reliable and valid instruments. Data were collected immediately postpartum, at 1 month postpartum and at 3 months postpartum. The general linear model was used to compare the groups for postpartum percentage changes in the outcome variables at first and third months, and the linear mixed model was used to compare the trend over the study period.

**Results:**

There was a statistically significant difference in Edinburgh Postnatal Depression Scale scores (*d*=–2.11; 95% CI −4.0 to −0.3; *P*=.03) between the intervention and control groups at 3 months postpartum after adjusting for covariates. The intervention group had a significant change over time compared with the control group.

**Conclusions:**

The technology-based PIP was found to be effective in reducing the risk of PND among new mothers and showed a generally positive trend in reducing PNA and loneliness and increasing perceived social support. This study highlights the importance of training paraprofessionals to provide needed support for new mothers postpartum. A further long-term evaluation of the PIP on maternal and family outcomes and its cost-effectiveness is needed to inform clinical practices.

**Trial Registration:**

ISRCTN Registry ISRCTN14864807; https://www.isrctn.com/ISRCTN14864807

**International Registered Report Identifier (IRRID):**

RR2-10.2196/resprot.9416

## Introduction

### Postnatal Depression

In a recent effort to improve women’s well-being globally, the World Health Organization has stepped up preventive efforts to reduce maternal morbidity and mortality [1] *.* As one of the leading causes of maternal morbidity [2], postnatal depression (PND) has an approximate global prevalence of 10% to 15% [3], with less than 1% of this population diagnosed with postpartum psychosis [4]. Common symptoms of PND include changes in appetite, insomnia, higher irritability, mood swings, anxiety [5], and even suicidal ideation in severe cases [6]. Women have also reported feelings of inadequacy [7,8], role conflicts [8,9], disconnection from others [6,9], loneliness [9], and dissatisfaction with life [7].

PND has garnered much attention because of its potential contribution to maternal mortality and its ripple effects on the family unit. According to a study by Goodman et al [10], maternal PND is the biggest risk factor for paternal PND, affecting 24% to 50% of all fathers, often jeopardizing marital relationships. Furthermore, PND adversely affects the quality of mother-child interaction and bonding [11] as mothers who suffer from PND tend to be more negligent, less tolerant, and hostile toward their children [12-14]. These tendencies not only impair the cognitive, behavioral, social-emotional development, and physical health of the child [14-17], but also increase their attachment anxiety [14], proneness to violence [18], and risks of psychopathology [14]. Therefore, early detections and preventions of PND are necessary to mitigating detrimental consequences at the individual and societal levels.

Despite its unspecified causes and suggested multifactorial etiologies [5,9], many studies have identified high-risk predictors of PND, including demographic, biological, psychological, obstetric, social, and lifestyle risk factors [9,19,20]. An in-depth analysis has also further revealed other risk factors that were derived from underlying unmet needs and social deficiencies experienced by postpartum mothers, such as the need for close, nonjudgmental confidants who empathize with them [9,21,22] and initiated support from others [5,23]. This indicates the vital role of social support during the postpartum period in reducing the risk of maternal PND.

### Importance of Social Support

Social support has long been proven to buffer stress and promote healthy psychological well-being [24]. This is especially crucial for new mothers during the stressful postpartum period. Although professional advice and informational support were much preferred by mothers [23], social support from partners and family members was shown to sustain mothers’ quality of life after childbirth and serve effectively as a buffer against PND [24]. However, Dennis et al [21] also stressed on the importance of support from other paraprofessionals such as experienced mothers. Other studies discovered that the sharing of experiences among mothers helps to develop a tight-knit community, which promotes a sense of belongingness, improves one’s sense of self-worth, boosts parenting confidence, and prevents PND [6,7,25]. This suggests that a support system involving sharing with another experienced mother who has undergone similar situations can potentially meet mothers’ needs in terms of empathy and having a nonjudgmental listening ear.

### Existing Technology-Based Interventions

Numerous studies identified help-seeking barriers among women at risk of psychological issues, namely lack of knowledge, practical barriers (eg, financial difficulties and work), and attitudinal barriers (eg, stigma) [21,26,27]. In a conservative multiracial country such as Singapore, traditional views and homebound confinement practices serve as additional help-seeking barriers. Therefore, technology-based interventions are an ideal alternative to increase local women’s accessibility to professional help and improve maternal outcomes [28,29]. With other advantages such as improved health care accessibility, flexibility, individualized care, and privacy [30], many randomized controlled trials have begun adopting technology-based supportive interventions [31-34].

Most of the existing literature has established the effectiveness of various technology-based interventions on maternal outcomes [31-33]. A Web-based study consisting of weekly Web educational sessions and phone calls from a coach was shown to decrease the risk of PND in 90% of the mothers at 6 months postpartum [32]. Another recent study utilizing telephone-based support provided by midwives was found to be effective in reducing the risk of PND in at-risk women at 8 and 12 weeks postpartum [33]. Similarly, a Canadian-based study [35] involving weekly telephone-based peer support was also found to reduce the risk of PND and postnatal anxiety (PNA) among at-risk mothers at 12 and 24 weeks postpartum. Despite encouraging results on maternal outcomes, these studies were mainly conducted in Western countries [34-36], required a health care professional [34], did not sample at-risk mothers [34,36], or did not have their interventions administered immediately postpartum [35,36]. Additionally, a study by Sjoberg et al [37] revealed that the new generation of mothers preferred online peer support over face-to-face or online consultations with health care professionals. Therefore, there is a need to adopt a technology-based approach and paraprofessional peer support to effectively meet the desires of new generation mothers in Singapore.

### Aim and Hypotheses

According to a recent review [30], an effective technology-based PND prevention intervention should be short term, be conducted immediately postpartum at an individual level, and target at-risk women instead of the general population. By incorporating all these elements, this study aims to examine the effectiveness of a technology-based peer-support intervention program (PIP) among mothers at risk of PND during the early postpartum period (3 months postpartum). The secondary maternal outcomes examined were PNA, loneliness, and perceived social support.

The hypothesis is that compared with the control group, mothers in the intervention group will report significantly lower scores for PND, PNA, and loneliness and higher scores for perceived social support at 3 months postpartum.

## Methods

### Study Design

The protocol of this study has been published [38]. The study was conducted from May 2017 to May 2018 at a local tertiary hospital, National University Hospital, in Singapore. This study adopted a randomized controlled, single-blinded 2-group pretest and posttest design. The research assistant who was responsible for data collection was blinded to the group allocation of the participants. Participants were randomized to the intervention and control groups using opaque envelopes containing nonduplicated numbers (1-138). A set of 69 numbers was generated from a research randomizer [39] to determine the allocation of the intervention group. Specific details on the randomization process can be found in the study protocol [38].

### Participants

Two samples of participants were recruited: (1) peer volunteers to facilitate the intervention program and (2) postnatal mothers at risk of PND. Peer volunteers were recruited through a blasting of emails to the study venue’s working community and by word of mouth based on the following inclusion criteria: (1) mothers who were aged at least 21 years, (2) proficient in verbal and written English, (3) delivered a healthy baby in the past, (4) had a self-reported history of and recovery from PND, (5) had a mobile phone and were willing to share their number and call needy mothers as instructed by the research team, and (6) planned to stay in Singapore for the next 6 months after recruitment to administer the peer-support intervention. Peer volunteers were excluded if they had any physical or mental conditions that interfered with their ability to participate in the study.

Mothers at risk of PND were recruited from the postnatal wards of a local tertiary hospital immediately postbirth based on the following inclusion criteria: (1) were aged at least 21 years, (2) could read and speak English, (3) owned a mobile phone and were willing to share their number, (4) planned to stay in Singapore for 3 months postbirth, (5) delivered a healthy baby without birth defects and/or medical complications, and (6) had a baseline Edinburgh Postnatal Depression Scale (EPDS) score of more than or equal to 9. Mothers were excluded if (1) they had a history of existing psychiatric illness, cognitive impairment, and/or major medical conditions that could interfere with their abilities to participate in the study and/or (2) had a vacuum- or forceps-assisted delivery with a fourth-degree perineal tear.

### Sample Size Calculation

On the basis of an independent sample *t* test to examine the differences between the control and intervention groups, assuming that there would be at least a medium Cohen effect size of 0.6 with 80% power and 0.05 significance level (2-sided), 47 participants were required in each group [40]. Factoring an attrition rate of 30%, a total of 138 participants (69 in each group) were recruited. A specification of the estimation of the effect size and the attrition rate is reported in the study protocol [38]. On the basis of a previous similar intervention study [35], 20 peer volunteers were recruited.

### Intervention

Mothers in the control group received standard routine postnatal care by the hospital, which included in-hospital care by an obstetrician, nurses, and a lactation consultant. Posthospital discharge, the only continuity of care provided, was in the form of appointments with obstetricians or neonatologists and breastfeeding hotline numbers. In addition to this standard postnatal care by the hospital, mothers in the intervention group received a technology-based peer-support program for 4 weeks postpartum. Before the recruitment of postnatal mothers, the peer volunteers underwent a half-a-day training session by a psychiatrist. The training session inculcated roleplaying and strategizing to hone skills required in administering successful technology-based peer support. Volunteers were also taught to conduct appropriate referrals to health care professionals, should the need arise. A training booklet was prepared and given to each peer volunteer for future references. The PIP intervention involved correspondence with a trained peer volunteer at least once a week (for 4 weeks) via phone calls, emails, or mobile communication applications (eg, WhatsApp), depending on each mother’s preference and convenience. During the introductory phone session, both sides shared their experience regarding emotional distress during the early postpartum period and extra efforts were made by the peer volunteer to build a strong relationship with the mother. Mothers were also informed that health care professionals would be notified if the mothers became too stressed during the correspondence. Subsequent sessions were individualized based on the unique needs of the mothers (eg, how to seek help from the family members and sharing one’s feelings with their partners). Peer volunteers were encouraged to keep a free text journal of their conversations, and the intensity and duration of each correspondence were recorded in an activity log. More specification on the peer volunteer training and intervention process can be found in the research protocol [38].

### Outcome Measures

The demographic data of the mothers were collected at the baseline using a self-reported questionnaire. Symptom scores for PND (primary outcome), PNA, loneliness score, and scores for perceived social support (secondary outcomes) were measured using a self-reported face-to-face questionnaire at the baseline and via Web-based questionnaires at the 4th and 12th week postpartum. The internal consistency of each instrument was measured using Cronbach alpha.

PND symptom score was measured using the 10-item EPDS [41]. The total score ranges from 0 to 30, with a higher score indicating a higher risk of PND. On the basis of previous trials [35,42], a recommended cut-off score of 9 was used to screen mothers at risk of PND and a score of more than 12 as a probable diagnosis for PND. The internal consistencies at baseline, 1 month postpartum, and 3 months postpartum were 0.59, 0.87, and 0.86, respectively.

The 9-item Patient Health Questionnaire (PHQ-9) [43] was extracted from the full PHQ used to diagnose and measure the severity of major depression. The total score ranges from 0 to 27, with a higher score indicating a higher severity of PND. The Cronbach alpha values for this study were 0.83, 0.86, and 0.92 for baseline, 1 month postpartum, and 3 months postpartum, respectively.

The State-Trait Anxiety Inventory (STAI) [44], a 40-item questionnaire using a 4-point Likert scale, was used to measure maternal anxiety. The total score ranges from 40 to 160, with a higher score suggesting a higher severity of anxiety. The STAI had high internal consistencies of 0.96, 0.97, and 0.98 for baseline, 1 month postpartum, and 3 months postpartum, respectively.

Loneliness score was measured using the 10-item University of California, Los Angeles Loneliness Scale (ULS) [45]. Items are rated on a 4-point Likert scale, with the total score ranging from 10 to 40. A higher score represents a higher level of loneliness. The ULS had high internal consistencies of 0.96, 0.97, and 0.97 at baseline, 1 month postpartum, and 3 months postpartum, respectively, in this study.

The Perceived Social Support for Parenting (PSSP) instrument developed by Leerkes and Crockenberg [46] was used to measure maternal satisfaction of the social support received from partners and others during the postpartum period. The instrument had a 5-point Likert scale and 2 4-item subparts: (1) social support received from the partner and (2) social support received from others. The total score ranges from 5 to 40, with a higher score implying a higher level of satisfaction of the received social support. The Cronbach alpha values for baseline, 1 month postpartum, and 3 months postpartum were 0.93, 0.89, and 0.92, respectively. Detailed descriptions of the instruments can be found in the study protocol [38].

### Data Collection

Nurses in the postnatal wards of the study hospital assisted in identifying suitable healthy mothers for the study. After being screened for eligibility, participants were given a thorough briefing on the study’s purpose and details. Mothers each signed a consent form upon agreeing to participate and proceeded to complete the EPDS questionnaire as a baseline measure. Mothers with EPDS scores of 9 and above were then randomly assigned to either the intervention or control group. Mothers in the intervention group were matched to a peer volunteer who contacted these mothers 2 to 3 days after their discharge from the hospital. Correspondence between peer volunteers and their paired mothers occurred at least once a week for 4 weeks via phone calls, emails, and mobile communication applications. Mothers in both the control and intervention groups concurrently proceeded with their standard hospital care and postnatal follow-ups. Weekly text reminders were sent by the research assistant nearing the 4th and 12th weeks to remind participants to complete the upcoming Web-based questionnaires. After 4 and 12 weeks, a research assistant who was blinded to the allocation of the participants forwarded a text message containing a Web link to the follow-up questionnaires to the participants and requested them to complete the questionnaires as soon as possible. An elaboration on the data collection process can be found in the research protocol [38].

### Data Analysis

All analyses were conducted using the IBM SPSS version 24.0 software (International Business Machines Corporation) with the statistical significance set at *P*<.05. The analysis was performed on the intention-to-treat population. Descriptive statistics were presented as mean (SD) and n (%) for continuous and categorical variables, respectively. A repeated measures analysis using a linear mixed model was used to assess the trend of the examined outcomes over the period of 3 months. The random effects were on subjects, and the fixed effects were on the following factors: age, marital status, antenatal class attendance, gender of infant, confinement period, and group and time interaction. The interaction effect of group and time was used to assess the differences over time. A general linear model was performed to compare the difference between intervention and control groups at 1 month and 3 months postpartum for the outcomes of EPDS, PSSP, ULS, and STAI by adjusting for the baseline. On the basis of previous literature [5,9,19,2], the following demographic factors were also adjusted in the general linear model for all outcomes: age, marital status, antenatal class attendance, gender of infant, and confinement period.

### Ethical Considerations

Ethics approval from the National Health Group Domain Specific Review Board (Ref number: NHG DSRB: 2017/00185) was obtained before the commencement of the study. All participants were briefed in detail on the research process before their written consents were obtained. Participation was strictly voluntary, and the participants were guaranteed anonymity and informed of their rights to withdraw at any time without consequences.

## Results

**Participants Data**

**Figure 1:**
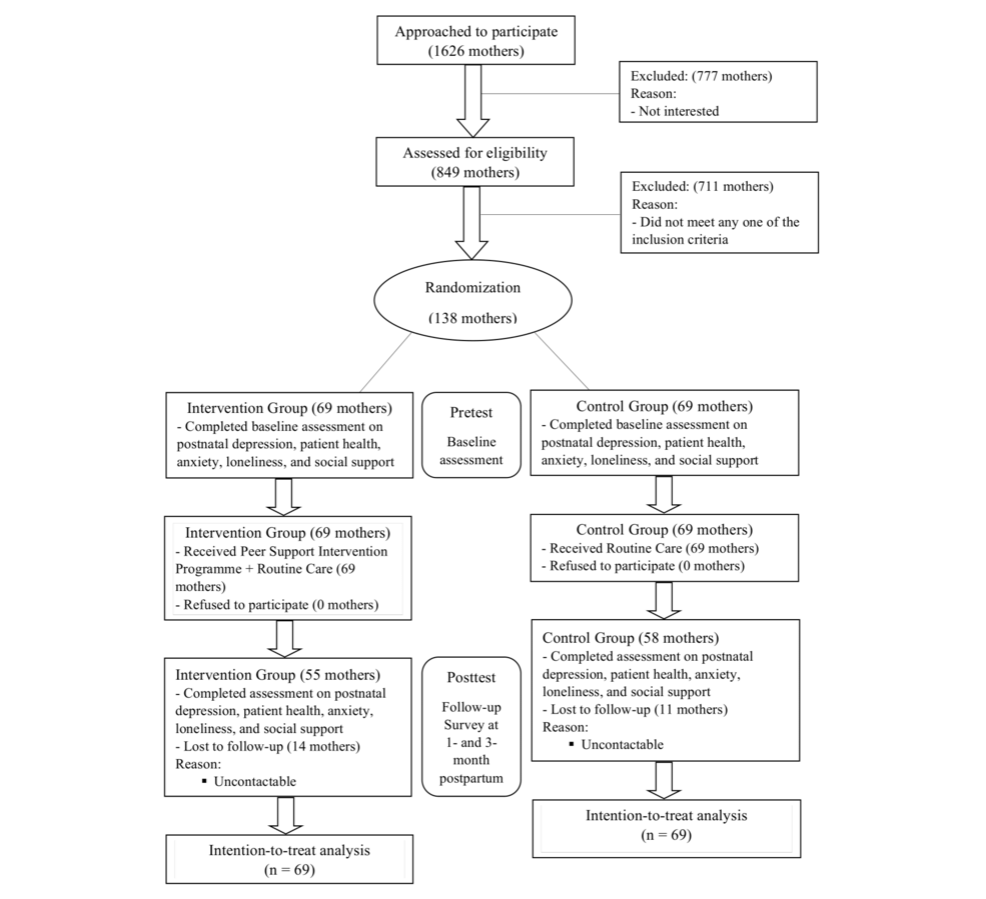
Consolidated Standards of Reporting Trials (CONSORT) flowchart of the study.

Figure 1 shows the Consolidated Standards of Reporting Trials flowchart of the study. A total of 138 mothers were recruited and randomized to the control (n=69) and intervention (n=69) groups. The baseline demographic data of all the participants are presented in Table 1. The participants had a mean age of 32.1 years (SD 4.35, range 23-43). Forty-two percent (53/138) of the participants were Chinese 96.4% (133/138) were married, 60.1% (83/138) had a university degree, and 67.6% (92/138) had a monthly household income of more than SGD $3000.

**Table 1:** Comparison of demographic characteristics between the 2 groups (N=138).

Characteristics		Total	Intervention group (n=69)	Control group (n=69)
Age (years), mean (SD); range		32.05 (4.35); 23-43	32.26 (3.90); 24-43	31.84 (4.77); 23-41
**Ethnicity, n (%)**				
	Chinese	58 (42.0)	30 (43)	28 (41)
	Malay	47 (34.1)	26 (38)	21 (30)
	Indian	16 (11.6)	5 (7)	11 (16)
	Others	17 (12.3)	8 (12)	9 (13)
**Marital status, n (%)**				
	Married	133 (96.4)	66 (96)	67 (97)
	Not married	5 (3.6)	3 (4)	2 (3)
**Highest education level, n (%)**				
	Secondary and below	15 (10.9)	9 (13)	6 (9)
	Preuniversity	40 (29.0)	17 (25)	23 (33)
	University	83 (60.1)	43 (62)	40 (58)
**Monthly household income (SGD $), n (%)**				
	<3000	44 (32.4)	24 (35)	20 (29)
	>$3000	92 (67.6)	44 (65)	48 (71)
**Attendance for prenatal course, n (%)**				
	Yes	40 (29.0)	26 (38)	14 (20)
	No	98 (71.0)	43 (62)	55 (80)
**Type of birth, n (%)**				
	Normal vaginal delivery	75 (54.7)	30 (43)	45 (66)
	Assisted delivery	14 (10.2)	11 (16)	3 (4)
	Cesarean section	48 (35.0)	28 (41)	20 (30)
**Baby’s gender, n (%)**				
	Twins	2 (1.4)	1 (1)	1 (1)
	Male	70 (50.8)	31 (45)	39 (57)
	Female	66 (47.8)	37 (54)	29 (42)
**Baby’s birth order, n (%)**				
	First	81 (58.7)	46 (67)	35 (51)
	Second	32 (23.2)	12 (17)	20 (29)
	Others	25 (18.1)	11 (16)	14 (20)
**First-time mother, n (%)**				
	Yes	81 (58.7)	46 (67)	35 (51)
	No	57 (41.3)	23 (33)	34 (49)
**Maternity leave, n (%)**				
	Yes	96 (69.6)	53 (77)	43 (62)
	No	42 (30.4)	16 (23)	26 (38)
**Confinement period, n (%)**				
	Yes	94 (87.0)	48 (89)	46 (85)
	No	14 (13.0)	6 (11)	8 (15)
**Baby’s feeding method, n (%)**				
	Breastfeeding	46 (42.2)	21 (39)	25 (45)
	Bottle feeding	6 (5.5)	3 (6)	3 (6)
	Both	57 (41.3)	30 (55)	27 (49)

Mothers mostly did not attend antenatal classes, had a normal vaginal delivery, followed a confinement period, and were first-time mothers. Follow-up assessments at 1 month postpartum were completed for all mothers in both the control (n=69) and intervention (n=69) groups. At 3 months postpartum, follow-up assessments were completed for 55 mothers from the intervention group (79%, 55/69) and 58 mothers from the control group (84%, 58/69). The overall attrition rate was 18.1%. As the intention-to-treat analysis was used, the outcome data for all 138 mothers were analyzed. Mothers who dropped out or did not provide outcome data at 3 months were still included in the main analysis (as the linear mixed model models data points rather than subjects). For sensitivity of the effect of the missing values on the results, a best-case and worst-case scenario was performed.

On the basis of previous literature, the outcome measures were adjusted for age, antenatal class attendance, gender of infant, and confinement period. Symptom scores for PND were measured using the EPDS and the PHQ*.*

The total scores for EPDS were lower in the intervention than the control group at both 1 month and 3 months postpartum. However, the difference of scores between groups was not statistically significant at 1 month even after adjusting for covariates (unadjusted: difference [*d*]=–0.91; 95% CI −2.5 to 0.6; *P*=.25; adjusted: *d*=−1.02; 95% CI −2.7 to 0.6; *P*=.23, but it was statistically significant at 3 months postpartum before and after adjustment (unadjusted: *d*=−1.77; 95% CI −3.5 to 0.0; *P*=.04; adjusted: *d*=−2.11; 95% CI −4.0 to −0.3; *P*=.03). On the basis of the linear mix model, there was also a statistically significant difference in the change of the total adjusted EPDS scores from baseline to 3 months postpartum for the intervention over the control group (*d*=−1.16; 95% CI −2.0 to −0.4; *P*=.004).

The total PHQ scores for the intervention group were lower than those of the control group at both 1 month and 3 months postpartum. At 1 month and 3 months, the unadjusted difference in scores between groups were statistically significant (first month: *d*=−1.80; 95% CI −3.3 to −0.3; *P*=.02; third month: *d*=−1.9; 95% CI −3.7 to −0.1; *P*=.04). However, the difference of scores between groups at both time points was no longer statistically significant after adjusting for covariates (first month: *d*=−1.59; 95% CI −3.3 to 0.1; *P*=.06; third month: *d*=−1.49; 95% CI −3.4 to 0.4; *P*=.11). Additionally, the difference in change of the total adjusted PHQ score for the intervention group from baseline to 3 months postpartum was also statistically significant (*d*=−1.00; 95% CI −1.9 to −0.1; *P*=.03).

At both 1 month and 3 months postpartum, the total STAI scores were lower in the intervention than the control group. The difference of scores between groups at 1 month and 3 months was not statistically significant even after adjusting for covariates (first month unadjusted: *d*=−3.63; 95% CI −10.7 to 3.5; *P*=.31; first month adjusted: *d*=−2.45; 95% CI −9.9 to 5.0; *P*=.52; 3 months unadjusted: *d*=−8.61; 95% CI −17.2 to 0.0; *P*=.05; 3 months adjusted: *d*=−7.89; 95% CI −16.4 to 0.7; *P*=.07). However, the difference in change of adjusted STAI scores for the intervention group was statistically significant across the 3 months (*d*=−4.16; 95% CI −7.9 to −0.4; *P*=.03).

The total scores for ULS were higher in the control group than in the intervention group at both 1 month and 3 months postpartum. At both time points, the unadjusted and adjusted mean differences of scores between groups were not statistically significant (first month unadjusted: *d*=−2.14; 95% CI −6.3 to 2.1; *P*=.32; first month adjusted: *d*=−2.45; 95% CI −7.0 to 2.1; *P*=.29; 3 months unadjusted: *d*=−3.90; 95% CI −8.2 to 0.4; *P*=.08; 3 months adjusted: *d*=−3.43; 95% CI −8.0 to 1.1; *P*=.14). There was also no statistically significant difference in change of ULS scores for the intervention group across 3 months even after adjustment (*d*=−2.16; 95% CI −4.4 to 0.0; *P*=.06).

At 1 month and 3 months postpartum, the control group had slightly higher PSSP scores than the control group. In addition, the difference of scores between groups was not statistically significant at both 1 month and 3 months even after adjustment (first month unadjusted: *d*=−2.11; 95% CI −5.2 to 1.0; *P*=.18; first month adjusted: *d*=−0.86; 95% CI −3.9 to 2.1; *P*=.57; 3 months unadjusted: *d*=1.09; 95% CI −2.9 to 5.1; *P*=.59; 3 months adjusted: *d*=−0.53; 95% CI −4.7 to 3.6; *P*=.80). The difference in change of total PSSP scores of the intervention group from baseline to 3 months postpartum was also not statistically significant (*d*=0.86; 95% CI −0.9 to 2.6; *P*=.33).

To account for potential type 1 error for multiple outcomes, the resultant *P* values were inflated by the number of outcomes analyzed, which is a factor of 5. Upon doing so, only the adjusted change of EPDS scores from baseline to the third month remained significant.

**Figure 2:**
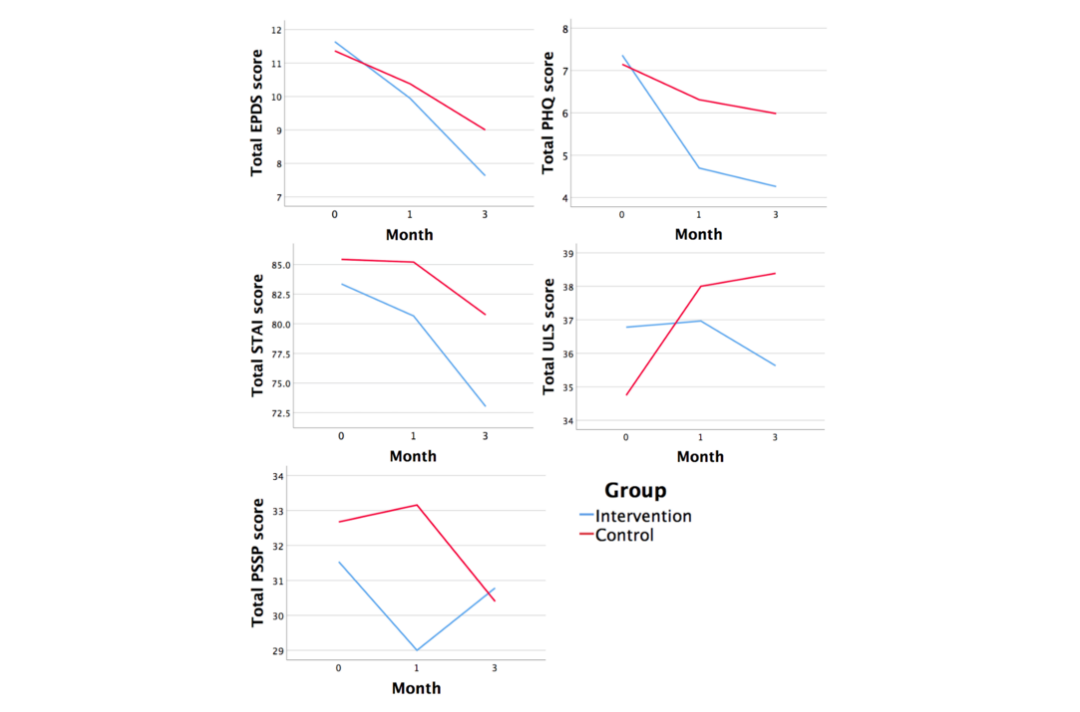
Trend comparison of mean outcome scores between groups across 3 months postpartum. EPDS: Edinburgh Postnatal Depression Scale; PHQ: Patient Health Questionnaire; PSSP: Perceived Social Support for Parenting; STAI: State-Trait Anxiety Inventory; ULS: University of California, Los Angeles Loneliness Scale.

Table 2 shows the changes in scores for all outcome variables, at each time point, including scores that were not adjusted for, whereas Table 3 shows the differences in change of outcome scores in the intervention group (over the control group) across 3 months. Although a change in ULS and PSSP scores between groups was not statistically significant across 3 months postpartum, Figure 2 shows good overall trending for all maternal outcomes from the baseline to 3 months postpartum. On the basis of the graph, mothers who received the intervention had better maternal outcome scores than the control group by the end of the third month.

A sensitivity analysis was also performed using the best and worst scores to replace missing values. *P* values from the best-case and worst-case analysis were then compared with the *P* values in the main analysis. The best-case analysis showed similar significant results as the main analysis. Sensitivity results are attached in Multimedia Appendices 1 and 2.

**Table 2:** Change in outcome scores between the intervention (I) and control (C) groups among mothers at 1 month and 3 months postpartum based on the general linear model.

Outcome variable	1 month								3 months							
	I, mean (SD), range	n^a^	C, mean (SD), range	n^a^	Unadjusted		Adjusted^b^		I, mean (SD), range	n^a^	C, mean (SD), range	n^a^	Unadjusted		Adjusted^b^	
					I–C (95% CI)	*P*	I–C (95% CI)	*P*					I–C (95% CI)	*P*	I–C (95% CI)	*P*
Postpartum depression (EPDS^c^)	11.4 (2.0), 7.5 to 15.3	56	12.4 (2.1), 8.4 to 16.5	58	−0.91 (−2.5 to 0.6)	.25	−1.02 (−2.7 to 0.6)	.23	9.8 (2.2), 5.5 to 14.3	54	12.0 (2.3), 7.5 to 16.5	57	−1.77 (−3.5 to 0.0)	.04^d^	−2.11 (−4.0 to −0.3)	.03^d^
Postpartum depression (PHQ^e^)	4.6 (2.0), 0.6 to 8.7	56	6.2 (2.1), 2.1 to 10.4	58	−1.80 (−3.3 to −0.3)	.02^d^	−1.59 (−3.3 to 0.1)	.06	5.5 (2.2), 1.0 to 10.0	54	7.0 (2.3), 2.4 to 11.6	57	−1.90 (−3.7 to −0.1)	.04^d^	−1.49 (−3.4 to 0.4)	.11
Postpartum anxiety (STAI^f^)	85.2 (9.9), 65.4 to 104.9	52	87.6 (9.9), 67.8 to 107.4	53	−3.63 (−10.7 to 3.5)	.31	−2.45 (−9.9 to 5.0)	.52	79.8 (11.3), 57.4 to 102.3	50	87.7 (11.3), 65.3 to 110.2	52	−8.61 (−17.2 to 0.0)	.05	−7.89 (−16.4 to 0.7)	.07
Loneliness (ULS^g^)	41.6 (5.5), 30.7 to 52.4	54	44.0 (5.6), 32.8 to 55.2	57	−2.14 (−6.3 to 2.1)	.31	−2.45 (−7.0 to 2.1)	.29	39.5 (5.4), 28.7 to 50.3	53	42.9 (5.6), 31.8 to 54.1	56	−3.90 (−8.2 to 0.4)	.08	−3.43 (−8.0 to 1.1)	.14
Perceived social support (PSSP^h^)	32.3 (2.6), 27.2 to 37.5	20	33.2 (2.6), 28.0 to 38.4	31	−2.11 (−5.2 to 1.0)	.18	−0.86 (−3.9 to 2.1)	.57	35.4 (4.4), 26.5 to 44.3	20	35.9 (4.3), 27.2 to 44.6	27	1.09 (−2.9 to 5.1)	.59	−0.53 (−4.7 to 3.6)	.78

^a^n values for adjusted analysis.

^b^Adjusted estimates were obtained from general linear models after being adjusted for baseline, age, marital status, antenatal class attendance, baby’s gender, and confinement period.

^c^EPDS: Edinburgh Postnatal Depression Scale.

^d^Significant *P* value <.05.

^e^PHQ: Patient Health Questionnaire.

^f^STAI: State-Trait Anxiety Inventory.

^g^ULS: University of California, Los Angeles Loneliness Scale.

^h^PSSP: Perceived Social Support for Parenting.

**Table 3:** Differences in the change of outcome scores across 3 months between intervention and control groups based on a linear mixed model.

Outcome variable	Trend difference (reference control group)			
	Unadjusted estimate (95% CI)	*P* value	Adjusted estimate^a^ (95% CI)	*P* value
Postpartum depression (EPDS^b^)	−0.90 (−1.7 to −0.6)	.02^c^	−1.16 (−2.0 to −0.4)	.004^b^
Postpartum depression (PHQ^d^)	−1.02 (−1.9 to −0.2)	.02^c^	−1.00 (−1.9 to −0.1)	.03^b^
Postpartum anxiety (STAI^e^)	−3.17 (−6.9 to 0.6)	.09	−4.16 (−7.9 to −0.4)	.03^b^
Loneliness (ULS^f^)	−2.19 (−4.3 to −0.1)	.04	−2.16 (−4.4 to 0.0)	.05
Perceived social support (PSSP^g^)	0.47 (−1.2 to 2.2)	.58	0.86 (−0.9 to 2.6)	.33

^a^Adjusted estimates were obtained from linear mixed models after being adjusted for baseline, age, marital status, antenatal class attendance, baby’s gender, and confinement period.

^b^EPDS: Edinburgh Postnatal Depression Scale.

^c^Significant *P* value <.05.

^d^PHQ: Patient Health Questionnaire.

^e^STAI: State-Trait Anxiety Inventory.

^f^ULS: University of California, Los Angeles Loneliness Scale.

^g^PSSP: Perceived Social Support for Parenting.

## Discussion

**Evaluation of Findings**

This study examined the effectiveness of a technology-based PIP among mothers at risk of PND. According to the score trend, the intervention group scored better than the control group for all maternal outcomes at both 1 month and 3 months postpartum, but only the difference in the EPDS scores between groups was shown to be statistically significant. This suggests that compared with mothers who only received routine hospital care, the PIP was generally effective in reducing the risks of PND, PNA, and loneliness and in increasing perceived social support received by the end of 3 months postpartum. Mothers further expressed their satisfaction with the intervention in a separate qualitative interview [47].

Although scores between groups were only significant for the EPDS, the EPDS and PHQ scores of the intervention group were still observably lower than those of the control group at 3 months postpartum, indicating the effectiveness of the peer-support program in reducing PND among at-risk mothers. The results were similar to previous studies [35,36] in which telephone-based peer support was found to reduce PND among depressed and at-risk mothers at 3 months postpartum. Our results are also constant with studies linking higher risks of PND to reduce perceived social support, few social networks, and close relationships [20,42,48]. Additionally, the study’s results correspond with large-scale reviews [30,49] that reported how short-term, individually based, technology-based interventions targeting at-risk women that were conducted immediately postpartum were the most effective in promoting positive maternal outcomes.

Many studies also established the high correlation and comorbidity between PND and PNA [50,51]; therefore, improvements in PND scores may inevitably lead to improvements in PNA scores. This corresponds with our results, which showed a significant change of EPDS, PHQ, and STAI scores across 3 months between groups. Similar results were found in Dennis et al’s peer-support study [35] where a positive trend in favor of the intervention group was found for maternal PNA. However, little empirical attention has been given to maternal anxiety as a standalone disorder [52]; hence, further research is required to examine effective preventive measures against PNA.

From our results, another notable observation was the sudden drop in PNA scores for both groups between 1 month and 3 months postpartum compared with the first month. Despite the cessation of the PIP at 1 month, it is possible that, by then, parents would have adapted to their new roles and gained sufficient parenting confidence and enhanced self-efficacy, which, in turn, reduced the risks of PNA. This is supported by a Finnish study [53], which reported that having high maternal parenting efficacy scores at 1 week postpartum reduces PNA at 1 month postpartum, and Kohlhoff’s study [54], which revealed an inverse correlation between parenting self-efficacy and PNA and PND. Another study [55] reporting the long-term sustainability of parental self-efficacy after the termination of a 6-week intervention could also be a plausible explanation to the sudden improvement in PNA scores in this study. Owing to the lack of studies examining the direct effects of paraprofessional peer support on parenting self-efficacy, further research is needed to validate these findings.

In terms of loneliness, there was an increase in loneliness scores for both groups from baseline to 1 month postpartum, with the control group having a steeper increase than the intervention group. Although loneliness scores continue to increase for mothers in the control group from 1 month to 3 months, loneliness scores for mothers in the intervention group decreased. This is evident that although the PIP was not able to fully relieve the sense of loneliness among mothers during the postpartum period, it still buffered mothers against loneliness compared with those who did not receive the PIP. A similar result was also noted in Dennis et al’s study [35], where although no positive trend was noted for loneliness at 3 months postpartum, the change in PND scores was significant. This conflicts with most literature that reported the predictive effects of loneliness on PND [3,20]. The trend observation was supported by a study that adopted a video conference method to examine loneliness [34]. Mothers reported that online face-to-face interactions were almost equivalent to having a physical presence and it facilitated rapport building [34]. This feature was lacking in our study. Furthermore, other face-to-face peer-support intervention studies also reported reduced feelings of loneliness among mothers [6,56]. This indicates the importance of a face-to-face element in technology-based support programs, which can better facilitate the sharing of experiences and alleviate feelings of loneliness. Another plausible cause is that at the end of the confinement period at 1 month, mothers were no longer physically isolated and could actively seek out family and friends for accompaniment [57]. Although the effectiveness of the PIP in mitigating loneliness might not be equivalent to the physical presence of a family member or friend, it can prevent the escalation of loneliness in mothers during the confinement period [35].

Mothers who did not receive peer support perceived a slight increase in social support at 1 month and a drastic decline in social support at 3 months postpartum, whereas mothers who received peer support only perceived a decrease in social support upon the termination of the intervention at 1 month to 3 months postpartum. This indicates the long-term effectiveness of the PIP in providing mothers with social support and that the termination of the program is a loss of an important source of social support to them. Over reliance on the PIP may result in a sudden drop in scores at 1 month, but the PIP also equipped them with help-seeking skills that might have caused an increase in scores at the third month. These findings correspond with other peer-support studies that reported an increased sense of perceived support in the intervention group [31,58] and studies linking the lack of social support to increased depressive symptoms [5,9,59]. However, our results conflict with other studies that reported an inverse correlation between social support and loneliness [60-62]. High social support scores of mothers in the control group for the first month could be due to the availability of instrumental and emotional support by family members, a partner, or a confinement nanny during the confinement period, whereas the decrease in scores after one month can be attributed to the end of paternal leave and the confinement period. During this time, mothers lose instrumental support from their partners and are abruptly entrusted with infant care responsibilities, which may result in an overwhelming sense of loss [63]. Social support is a three-dimensional construct consisting of emotional, informational, and instrumental support [19]. Peer-support interventions may fulfill the emotional aspect and, to a certain extent, informational support, but mothers reported higher needs for instrumental support during the postpartum period [23]. Therefore, for optimal outcomes, the PIP should be administered concurrently with instrumental help to provide well-rounded social support to mothers.

### Limitations and Recommendation for Future Studies

To the best of our knowledge, this is the first technology-based peer-support study based in Asia that showed a preventive effect against PND. Therefore, it is a valuable contribution to ongoing region-specific research on the prevention of PND. However, a major limitation of this study is that it was a single-site study targeting only English-speaking mothers. Future studies can consider integrating more languages to cater to minority groups. Another limitation is that the intervention was only administered during the postpartum period. Considering that antenatal depression is a main predictor of PND, future studies should examine the effectiveness of such interventions during the perinatal period. Additionally, maternal outcomes included in this study were limited and infant outcomes were lacking. Given that most maternal outcomes are interrelated, other outcomes such as parenting self-efficacy and parenting satisfaction as well as an evaluation of the effectiveness of the program on infant development can be included in subsequent studies. Finally, an evaluation of the cost-effectiveness of the PIP will provide a holistic view on the effectiveness of this intervention.

### Conclusions

This randomized controlled trial demonstrated the effectiveness of a technology-based peer-support program in reducing maternal PND. Besides receiving standard postnatal care, additional participation in the PIP was shown to improve the general well-being of mothers at the end of 3 months postpartum. This study adds value to the use of technology and trained paraprofessionals in combating PND among new mothers. Although future rigorous trials are needed to evaluate the effectiveness of the PIP further, health care professionals can involve paraprofessionals such as family members in supporting new mothers during the stressful postpartum period. This may enhance not only maternal outcomes but also the future well-being of the family, thus creating positive childbirth experiences for mothers.

## Appendix

Multimedia Appendix 1 Sensitivity analysis (best-case and worst-case scenario) using the general linear model.

Multimedia Appendix 2 Sensitivity analysis (best-case and worst-case scenario) using the linear mixed model.

Multimedia Appendix 3 CONSORT‐EHEALTH checklist (V 1.6.1).

## Abbreviations

EPDS: Edinburgh Postnatal Depression Scale
PHQ: Patient Health Questionnaire
PIP: peer-support intervention program
PNA: postnatal anxiety
PND: postnatal depression
PSSP: Perceived Social Support for Parenting
STAI: State-Trait Anxiety Inventory
ULS: University of California, Los Angeles Loneliness Scale
