# Supplementary material for: Evaluation of a Technology-Based Peer-Support Intervention Program for Preventing Postnatal Depression (Part 1): Randomized Controlled Trial
Source: J Med Internet Res. 2019 Aug 29;21(8):e12410. doi: 10.2196/12410 (PMC6744221; doi:10.2196/12410)
Supplement: Multimedia Appendix 1 [file jmir_v21i8e12410_app1.docx]

**Appendix 1a.** Best case scenario: Change in outcome scores between the intervention and control groups amongst mothers at first and third month postpartum based on General linear model.

|  | One month | | | | | | | | Three months | | | | | | | |
| --- | --- | --- | --- | --- | --- | --- | --- | --- | --- | --- | --- | --- | --- | --- | --- | --- |
| Outcome variable |  |  |  |  | Unadjusted | | Adjusted | |  |  |  |  | Unadjusted | | Adjusted | |
|  | Intervention (I)  Mean (SD), range | n^**^ | Control (C)  Mean (SD), range | n^**^ | I-C [95% CI] | *P* | I-C [95% CI] | *P* | Intervention (I)  Mean (SD), range | n^**^ | Control (C)  Mean (SD), range | n^**^ | I-C [95% CI] | *P* | I-C [95% CI] | *P* |
| Postpartum depression (EPDS) | 11.3 (2.2),  7.1 to 15.7 | 56 | 12.5 (2.2),  8.1 to 17.0 | 58 | -0.81  [-2.7, 1.0] | .389 | -1.16  [-2.9, 0.6] | .193 | 11.4 (2.2),  7.1 to 15.7 | 54 | 12.5 (2.2),  8.1 to 17.0 | 57 | -1.59  [-3.4, 0.2] | .083 | -1.16  [-2.9, 0.6] | .193 |
| Postpartum depression (PHQ) | 4.7 (2.0),  0.7 to 8.8 | 56 | 6.2 (2.1),  2.0 to 10.3 | 58 | -1.60  [-3.0, -0.2] | .030* | -1.45  [-3.1, 0.2] | .079 | 4.7 (2.0),  0.7 to 8.8 | 54 | 6.2 (2.1),  2.0 to 10.3 | 57 | -1.74  [-3.3, -0.1] | .033* | -1.45  [-3.1, 0.2] | .079 |
| Postpartum anxiety (STAI) | 81.6 (9.4),  62.9 to 100.2 | 52 | 85.5 (9.7),  66.2 to 14.8 | 53 | -5.08  [-13.1, 2.9] | .212 | -3.93  [-11.4, 3.6] | .302 | 81.6 (9.4),  62.9 to 100.2 | 50 | 85.5 (9.7),  66.2 to 104.8 | 52 | -7.89  [-16.2, 0.4] | .063 | -3.93 | .302 |
| Loneliness (ULS) | 42.0 (5.6),  30.9 to 53.2 | 54 | 43.8 (5.8),  32.4 to 55.3 | 57 | -2.24  [-6.5, 2.0] | .300 | -1.84  [-6.3, 2.6] | .417 | 42.0 (5.6),  30.9 to 53.2 | 53 | 43.8 (5.8),  32.4 to 55.3 | 56 | -3.95  [-8.2, 0.3] | .071 | -1.84  [-6.3, 2.6] | .417 |
| Perceived social support (PSSP) | 37.0 (2.6),  31.9 to 42.1 | 20 | 36.9 (2.6),  31.7 to 42.1 | 31 | -0.02  [-1.8, 1.8] | .984 | 0.12  [-1.8, 2.0] | .904 | 37.0 (2.6),  31.9 to 42.1 | 20 | 36.9 (2.7),  31.7 to 42.1 | 27 | 1.53  [-0.5, 3.5] | .131 | 0.12  [-1.8, 2.0] | .904 |

^*^ Significant *P*-value<0.05.

^**^n values for adjusted analysis
Adjusted estimates were obtained from general linear models after adjusted for baseline, age, marital status, antenatal class attendance, baby’s gender, and confinement period. 95% CI=95% Confidence interval, C=Control, EPDS=Edinburgh Postnatal Depression Scale, I=Intervention, PHQ=Patient Health Questionnaire, PSSP=Perceived Social Support for Parenting, SD=Standard deviation, STAI=State-Trait Anxiety Inventory, ULS=University of California, Los Angeles Loneliness Scale

**Appendix 1b.** Worst case scenario: Change in outcome scores between the intervention and control groups amongst mothers at first and third month postpartum based on General linear model.

|  | One month | | | | | | | | Three months | | | | | | | |
| --- | --- | --- | --- | --- | --- | --- | --- | --- | --- | --- | --- | --- | --- | --- | --- | --- |
| Outcome variable |  |  |  |  | Unadjusted | | Adjusted | |  |  |  |  | Unadjusted | | Adjusted | |
|  | Intervention (I)  Mean (SD), range | n^**^ | Control (C)  Mean (SD), range | n^**^ | I-C [95% CI] | *P* | I-C [95% CI] | *P* | Intervention (I)  Mean (SD), range | n^**^ | Control (C)  Mean (SD), range | n^**^ | I-C [95% CI] | *P* | I-C [95% CI] | *P* |
| Postpartum depression (EPDS) | 11.3 (3.1),  5.2 to 17.4 | 56 | 11.5 (3.2),  5.2 to 17.8 | 58 | 0.03  [-2.8, 2.9] | .984 | -0.27  [-2.8, 2.2] | .226 | 9.9 (3.8),  2.4 to 17.4 | 54 | 10.9 (3.9),  3.1 to 18.6 | 57 | -0.31  [-3.6, 2.9] | .852 | -0.98  [-4.0, 2.1] | .529 |
| Postpartum depression (PHQ) | 5.0 (3.5),  -2.0 to 12.0 | 56 | 5.9 (3.6),  -1.3 to 13.1 | 58 | -0.67  [-4.1, 2.8] | .702 | -0.86  [-3.7, 1.9] | .544 | 6.0 (4.3),  -2.5 to 14.5 | 54 | 6.4 (4.4),  -2.3 to 15.2 | 57 | -0.36  [-4.1, 3.4] | .850 | -0.41  [-3.8, 3.0] | .113 |
| Postpartum anxiety (STAI) | 80.2 (12.4),  55.5 to 104.9 | 52 | 81.8 (12.8),  56.4 to 107.3 | 53 | -1.38  [-13.0, 10.2] | .815 | -1.61  [-11.5, 8.3] | .748 | 76.0 (15.3),  45.7 to 106.3 | 50 | 80.0 (15.7),  48.7 to 111.2 | 52 | -2.49  [-15.6, 10.7] | .709 | -3.96  [-16.1, 8.2] | .520 |
| Loneliness (ULS) | 42.7 (7.6),  27.6 to 57.7 | 54 | 43.6 (7.8),  28.0 to 59.1 | 57 | -3.12  [-6.4, 0.2] | .062 | -0.90  [-7.0, 5.2] | .769 | 40.5 (8.4),  23.7 to 57.2 | 53 | 42.1 (8.7),  24.8 to 59.3 | 56 | -1.41  [-8.5, 5.6] | .692 | -1.59  [-8.3, 5.1] | .641 |
| Perceived social support (PSSP) | 25.1 (4.4),  16.4 to 33.9 | 20 | 28.4 (4.5),  19.4 to 37.3 | 31 | -0.27  [-2.8, 2.2] | .829 | -3.25  [-6.6, 0.1] | .566 | 28.8 (4.4),  20.1 to 37.5 | 20 | 30.4 (4.5),  21.4 to 39.3 | 27 | -1.23  [-4.5, 2.0] | .457 | -1.57  [-4.9, 1.8] | .353 |

^*^ Significant *P*-value<0.05.

^**^n values for adjusted analysis
Adjusted estimates were obtained from general linear models after adjusted for baseline, age, marital status, antenatal class attendance, baby’s gender, and confinement period. 95% CI=95% Confidence interval, C=Control, EPDS=Edinburgh Postnatal Depression Scale, I=Intervention, PHQ=Patient Health Questionnaire, PSSP=Perceived Social Support for Parenting, SD=Standard deviation, STAI=State-Trait Anxiety Inventory, ULS=University of California, Los Angeles Loneliness Scale
