# Supplementary material for: Evaluation of a Technology-Based Peer-Support Intervention Program for Preventing Postnatal Depression (Part 1): Randomized Controlled Trial
Source: J Med Internet Res. 2019 Aug 29;21(8):e12410. doi: 10.2196/12410 (PMC6744221; doi:10.2196/12410)
Supplement: Multimedia Appendix 2 [file jmir_v21i8e12410_app2.docx]

**Appendix 2a.** Best case scenario: Change in outcome scores within control and intervention groups across three months based on linear mixed model.

|  | Trend difference (ref Control group) | | | |
| --- | --- | --- | --- | --- |
| Outcome variable | Unadjusted Estimate  [95% CI] | *P* | Adjusted Estimate [95% CI] | *P* |
| Postpartum depression (EPDS) | -0.87  [-1.7, -0.0] | .044* | -1.16  [-2.0, -0.3] | .006* |
| Postpartum depression (PHQ) | -0.93  [-1.7, -0.1] | .025* | -0.93  [-1.8, -0.0] | .040* |
| Postpartum anxiety (STAI) | -3.98  [-7.9, -0.1] | .044* | -4.57  [-8.3, -0.8] | .017* |
| Loneliness (ULS) | -2.33  [-4.4, -0.2] | .029* | -1.95  [-4.1, 0.2] | .078 |
| Perceived social support (PSSP) | 1.28  [0.2, 2.4] | .020* | 1.13  [-0.1, 2.3] | .067 |

^*^ Significant *P*-value<0.05.

Adjusted estimates were obtained from linear mixed models after adjusted for baseline, age, marital status, antenatal class attendance, baby’s gender, and confinement period. 95% CI=95% Confidence interval, C=Control, EPDS=Edinburgh Postnatal Depression Scale, I=Intervention, PHQ=Patient Health Questionnaire, PSSP=Perceived Social Support for Parenting, STAI=State-Trait Anxiety Inventory, ULS=University of California, Los Angeles Loneliness Scale

**Appendix 2b.** Worst case scenario: Change in outcome scores within control and intervention groups across three months based on linear mixed model.

|  | Trend difference (ref Control group) | | | |
| --- | --- | --- | --- | --- |
| Outcome variable | Unadjusted Estimate  [95% CI] | *P* | Adjusted Estimate [95% CI] | *P* |
| Postpartum depression (EPDS) | -0.22  [-1.6, 1.1] | .749 | -0.89  [-2.1, 0.3] | .156 |
| Postpartum depression (PHQ) | -0.25  [-1.9, 1.4] | .760 | -0.65  [-2.1, 0.8] | .388 |
| Postpartum anxiety (STAI) | -1.17  [-6.8, 4.5] | .682 | -3.27  [-8.4, 1.8] | .206 |
| Loneliness (ULS) | -1.38  [-4.6, 1.8] | .394 | -1.84  [-4.9, 1.2] | .240 |
| Perceived social support (PSSP) | -0.35  [-2.1, 1.4] | .702 | -0.28  [-2.2, 1.6] | .775 |

^*^ Significant *P*-value<0.05.

Adjusted estimates were obtained from linear mixed models after adjusted for baseline, age, marital status, antenatal class attendance, baby’s gender, and confinement period. 95% CI=95% Confidence interval, C=Control, EPDS=Edinburgh Postnatal Depression Scale, I=Intervention, PHQ=Patient Health Questionnaire, PSSP=Perceived Social Support for Parenting, STAI=State-Trait Anxiety Inventory, ULS=University of California, Los Angeles Loneliness Scale
